# Bivalent Copper Ions Promote Fibrillar Aggregation of KCTD1 and Induce Cytotoxicity

**DOI:** 10.1038/srep32658

**Published:** 2016-09-06

**Authors:** Zhepeng Liu, Feifei Song, Zhi-li Ma, Qiushuang Xiong, Jingwen Wang, Deyin Guo, Guihong Sun

**Affiliations:** 1 School of Basic Medical Sciences, Wuhan University, Wuhan 430072, P.R. China

## Abstract

Potassium channel tetramerization domain containing 1 (KCTD1) family members have a BTB/POZ domain, which can facilitate protein-protein interactions involved in the regulation of different signaling pathways. KCTD proteins have potential Zn^2+^/Cu^2+^ binding sites with currently unknown structural and functional roles. We investigated potential Cu^2+^-specific effects on KCTD1 using circular dichroism, turbidity measurement, fluorescent dye binding, proteinase K (PK) digestion, cell proliferation and apoptosis assays. These experiments indicate that the KCTD1 secondary structure assumes greater β-sheet content and the proteins aggregate into a PK-resistant form under 20 μM Cu^2+^, and this β-sheet-rich aggregation with Cu^2+^ promotes fibril formation, which results in increased cell toxicity by apoptosis. Our results reveal a novel role for Cu^2+^ in determining the structure and function of KCTD1.

Metal ions have essential roles in human physiology^1^,^2^, and many proteins have greater binding affinity for Cu^2+^ than other divalent metal ions^3^. Copper has a wide intracellular distribution and essential biofunctions, but it is toxic when in excess. Copper is the third-most abundant metal in brain tissue^4^. Copper levels accumulate in diseased human tissues and are associated with plaque formation of prions, Alzheimer’s, Parkinson’s, and Huntington’s^5^,^6^,^7^. These human diseases have been linked to protein misfolding and aggregation^8^,^9^,^10^. Copper acts as an important cofactor in changing protein secondary structure, and can accelerate or delay protein misfolding and aggregation rate *in vitro*^6^,^11^. Copper has been linked to the progression of protein-misfolding diseases^7^,^12^, which are characterized by the presence of fibrillar aggregates or amyloid plaques. Several studies have suggested that formation of amyloid deposits in neurological disorders results from increased levels of metal ions and subsequent failure of the ubiquitin-proteasomal system (UPS) of protein degradation^13^,^14^.

Biological investigations have demonstrated that exposure of the UPS to increasing amounts of metal ions affects its primary proteolytic activity, which suggests a close relationship between age-dependent increase in metal ion levels in the brain, UPS failure, and disease onset. Protein ubiquitination is usually mediated by diverse E3 ligase enzymes that serve as scaffolds for interactions with substrate-recruiting adaptor proteins. BTB-domain-containing KCTD proteins may act as adaptors for interaction between Cul3 ubiquitin ligase and its substrates. Recent work shows that KCTD proteins participate as substrate-specific adaptors in multimeric cullin-E3 ligase reactions by recruiting proteins for ubiquitination and subsequent degradation by the proteasome^15^,^16^,^17^. KCTD1 is a member of the KCTD protein family; it contains a bric-a-brac, tramtrack, and broad complex (BTB) domain. Recent studies of KCTD involvement in protein degradation yielded a surprisingly unifying view: KCTD1 enhances the ubiquitination/degradation of β-catenin in a concentration-dependent manner in HeLa cells^18^. Our previous study showed that KCTD1 interacted with the cellular prion protein (PrP^C^) with high affinity, and suggested that KCTD1 could serve as an adaptor that mediates PrP ubiquitination and degradation via cullin binding^19^. Copper accumulates in plaques in neurological disorders, and KCTD1 is strongly expressed in brain cells^20^. These results raise some interesting questions regarding copper ion regulation of KCTD1 structure and function. For example, can altered copper ion levels associated with protein misfolding diseases affect KCTD1 structure?

To address these questions, we performed a set of experiments to determine whether Cu^2+^ influences KCTD1 structure, and if so, how this occurs. We report that in the presence of Cu^2+^, KCTD1 tends to form aggregates in a manner that is dependent on the Cu^2+^ concentration. We show that KCTD1 aggregation in the presence of Cu^2+^ is fibrillar and resistant to proteinase K (PK) digestion. Furthermore, we show that KCTD1 aggregation in the presence of Cu^2+^ is functionally cytotoxic. Our studies suggest that Cu^2+^ may affect KCTD1 misfolding/aggregation and lead to cytotoxicity.

## Results

### Cu^2+^ alters the structure of KCTD1

**Figure 1:**
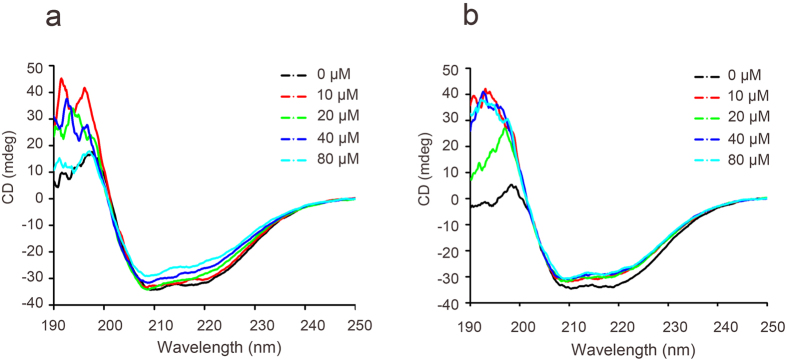
Cu^2+^ induces changes in KCTD1 secondary structure. Far-UV circular dichroism assay of 0.4 μg/μl KCTD1 incubated with the indicated concentrations (10, 20, 40, and 80 μM) of (**a**) Cu^2+^ or (**b**) Zn^2+^.

Approximately 24% of all proteins require metal ions for normal function^21^. KCTD1 contains a highly conserved BTB/POZ domain that binds metal ions. We investigated whether metal ions affect KCTD1 structure by monitoring the far-UV CD spectra of purified KCTD1 in the presence or absence of Cu^2+^/Zn^2+^. The secondary structure of purified KCTD1 in the absence of metal ions had spectral features typical of a protein containing significant alpha-helical structure, with well-defined minima at 208 and 222 nm. After 15 min incubation with varying Cu^2+^ concentrations, the troughs around 208 and 222 nm were reduced, which is indicative of an increase in β-sheet structure (Fig. 1a). However, the KCTD1 secondary structure did not change in the presence of equivalent Zn^2+^ concentrations (Fig. 1b). Therefore, we focused on determining how Cu^2+^ affects KCTD1 structure and function.

**Figure 2:**
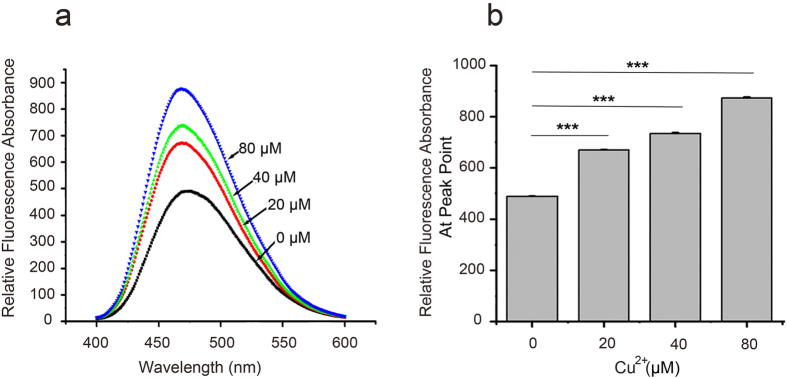
Analysis of ANS fluorescence emission indicates that Cu^2+^ enhances KCTD1 hydrophobicity. KCTD1 (0.4 μg/μl) was incubated in the presence or absence of Cu^2+^ (at pH 7.0) for 2 h at 37 °C. (**a**) Fluorescence spectra of KCTD1 with different Cu^2+^ concentrations (0, 20, 40, and 80 μM). (**b**) Peak fluorescence values at different Cu^2+^ concentrations as in (**a**). The data shown were the mean ± s.d. of 3 independent experiments. ***P < 0.001; t-test.

To further characterize Cu^2+^-mediated changes in KCTD1 structure, we used 8-anilino-1-naphthalenesulfonic acid, a naphthalene derivative that undergoes an increase in fluorescence emission on interaction with hydrophobic sites. The bis-ANS fluorescence emission was significantly higher in the sample containing Cu^2+^ than in control samples without Cu^2+^, and the increase was proportional to the Cu^2+^ concentration (Fig. 2a,b). These results were consistent with those of CD analysis and indicated that Cu^2+^ increased KCTD1 hydrophobicity.

### Cu^2+^ triggers KCTD1 aggregation

**Figure 3:**
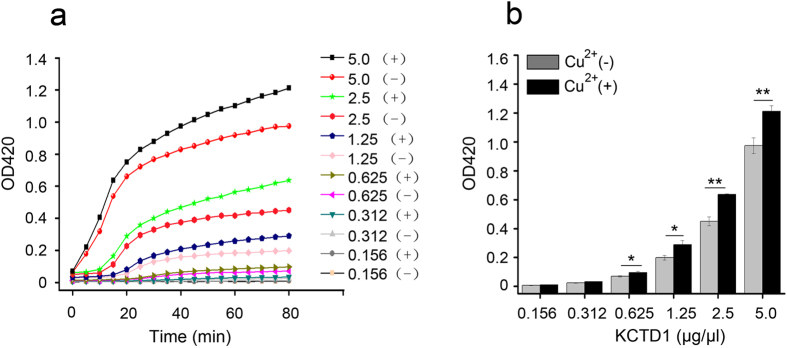
KCTD1 aggregates *in vitro* in a concentration-dependent manner using the turbidity assay. (**a**) Time-course aggregation of different KCTD1 concentrations (0.156−5.0 μg/μl) at pH 7.0 and 37 °C, in the presence (+) of 20 μM Cu^2+^, and in the absence (−) of 20 μM Cu^2+^. (**b**) The turbidity of different KCTD1 concentrations (0.156−5.0 μg/μl) were monitored in the presence (+) or absence (−) of Cu^2+^ after incubation for 80 min. The data shown were the mean ± s.d. of 3 independent experiments. *P < 0.05; **P < 0.01; t-test.

The fluorescence emission analysis showed that the interaction of Cu^2+^ with KCTD1 exposed a hydrophobic surface on the protein, which could promote protein aggregation. To test this, we measured the turbidity of KCTD1 after incubating at 37 °C for up to 80 min with and without Cu^2+^ by a all-in-one Microplate Reader at OD420. The results indicated that few KCTD1 oligomers were present at the beginning of the incubation in PBS containing 0 or 20 μM CuSO_4_. The turbidity increased over time during the 80 min incubation, and the amount of aggregates increased with increasing KCTD1 concentrations (Fig. 3a). Importantly, Cu^2+^ enhanced protein aggregation and increased turbidity (Fig. 3a,b).

### Cu^2+^ causes the formation of fibrillar KCTD1 aggregates

**Figure 4:**
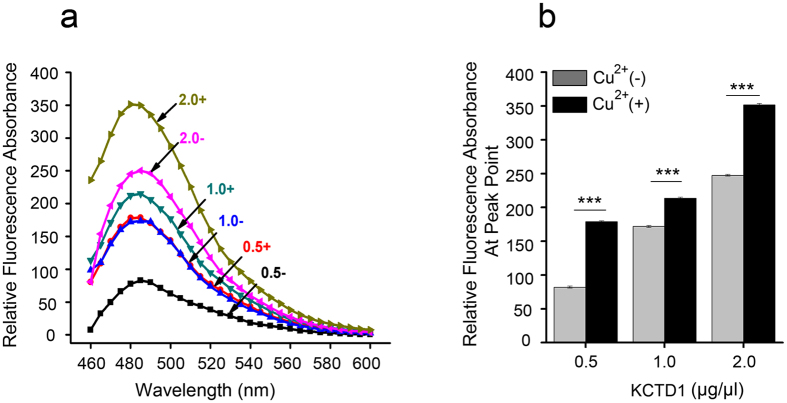
Thioflavin T binding analysis indicates that Cu^2+^ promotes fibrillar aggregation of KCTD1. KCTD1 concentrations of 0.5−2.0 μg/μl were incubated for 2 h (pH 7.0) in the presence (**+**) or absence (−) of 20 μM Cu^2+^. (**a**) Fluorescence spectra of KCTD1 with ThT. (**b**) Peak values of the spectra at different KCTD1 concentrations with ThT. The data shown were the mean ± s.d. of 3 independent experiments. ***P < 0.001; t-test.

To investigate the nature of Cu^2+^-mediated KCTD1 aggregation, we evaluated binding of the common dye ThT to KCTD1. The intensity of ThT fluorescence emission (around 485 nm) drastically increases on binding to fibrils containing β-sheet structures, such as amyloid fibrils^22^. Therefore, we utilized ThT to detect the formation of KCTD1 fibrils in the presence or absence of 20 μM CuSO_4_. There was a remarkable difference in ThT fluorescence intensity in samples with and without Cu^2+^ (Fig. 4a,b). ThT fluorescence intensity at peak point was much higher for KCTD1 in the presence of Cu^2+^ than that without Cu^2+^ (Fig. 4b), which was similar to that observed for amyloids (Fig. 4a). This result indicated that Cu^2+^ caused an increase in β-sheet formation, which caused KCTD1 aggregation and fibrillar formation.

**Figure 5:**
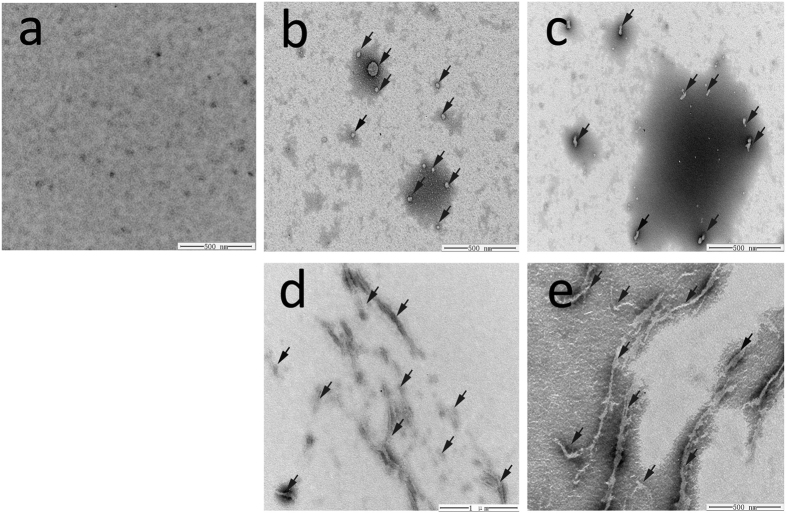
Morphology of KCTD1 aggregates reveals by transmission electron microscopy. KCTD1 was incubated for 2 hours at 37 °C with or without 20 μM Cu^2+^. (**a**) Freshly purified KCTD1 presented no aggregates. (**b**,**c**) KCTD1 incubated without Cu^2+^ formed aggregates with irregular morphologies. (**d,e**) KCTD1 incubated with Cu^2+^ formed fibrillar aggregates. Irregular aggregates and fibrillar aggregation in (**b**–**e**) were indicated with black arrow mark.

Next, we analyzed KCTD1 morphology in the presence or absence of Cu^2+^ by electron microscopy. In the absence of Cu^2+^, protein aggregates that formed after 30 min incubation were amorphous with irregular shapes (Fig. 5b,c), whereas no aggregates were detected before the incubation (Fig. 5a). By contrast, KCTD1 in the presence of Cu^2+^ formed numerous fibrils of variable lengths (Fig. 5d,e). These results indicated that Cu^2+^ induced the formation of KCTD1 fibrils.

### Cu^2+^-induced KCTD1 aggregates are more resistant to PK digestion

**Figure 6:**
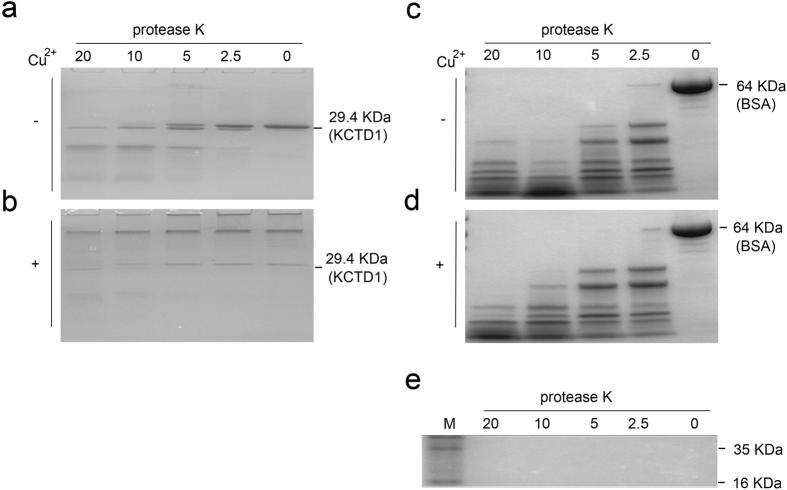
Cu^2+^-induced KCTD1 fibrillar aggregates have enhanced resistance to proteolytic digestion. KCTD1 (0.8 μg/μl) was incubated without (**a**) or with (**b**) 20 μM Cu^2+^ for 2 h at 37 °C, followed by digestion with 0–20 μg/ml proteinase K at 37 °C for 35 min. (**c**,**d**) as the same as (**a**,**b**) respectively, KCTD1 was replaced with bovine serum albumin (BSA). (**e**) Proteinase K was not detectable in the SDS–page gel at the concentrations used (0–20 μg/ml).

We determined whether KCTD1 aggregates could be cleaved by proteinase K. KCTD1 or BSA control were incubated with or without 20 μM CuSO_4_ for 2 hours at 37 °C. The protein samples were then digested with protease K for 35 min and quenched by 2 mM PMSF. And then the treated samples were analyzed by SDS-PAGE gel. It is distinctly different in Fig. 6a,b that the KCTD1 with Cu^2+^ had obvious the monomer and polymer; On the contrary, the polymer was indiscernible in the gel of the KCTD1 free Cu^2+^. KCTD1 fibrillar aggregates formed in the presence of Cu^2+^ were resistant to SDS-dissolution and PK digestion under a range of PK concentrations (2.5, 5, 10, and 20 μg/ml) (Fig. 6b), whereas KCTD1 aggregates formed in the absence of Cu^2+^ were no SDS-resistant and easily digested by PK in a concentration-dependent manner (Fig. 6a). Although, BSA control samples were treated in the same condition as KCTD1, however, the BSA control was digested by PK irrespective of the presence or absence of Cu^2+^, after digestion, the same bands were observed in the SDS-PAGE gel (Fig. 6c,d). Although PK molecular weight was about 29 KD, which makes it diffcult to distinguish with KCTD1 (molecular weight about 29.4 KD) in the SDS-PAGE gel, Fig. 6e showed the different concentrations PK (2.5, 5, 10, and 20 μg/ml) had no band from 25 to 35 KD in SDS- PAGE gel. And it also indicated the PK at experimental concentrations didn’t affect the bands of KCTD1 digestion.

### KCTD1 aggregation in the presence of Cu^2+^ is more cytotoxic to cell

**Figure 7:**
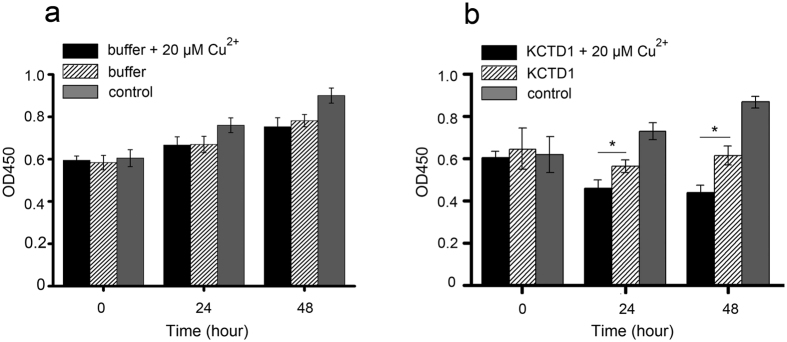
KCTD1 aggregates formed in the presence of Cu^2+^ are more cytotoxic than those formed in the absence of Cu^2+^. Cytotoxicity of KCTD1 aggregates formed in the present or absence of Cu^2+^ was determined by the CCK-8 assay on HuH7 cells versus untreated control. (**a**) Effect of sole Cu^2+^ on HuH7 cell line proliferation. (**b**) KCTD1 aggregates formed in the presence of Cu^2^^+^ induced cell toxicity on HuH7 cells, the data shown were the mean ± s.d. of 3 independent experiments. *P < 0.05; t-test.

**Figure 8:**
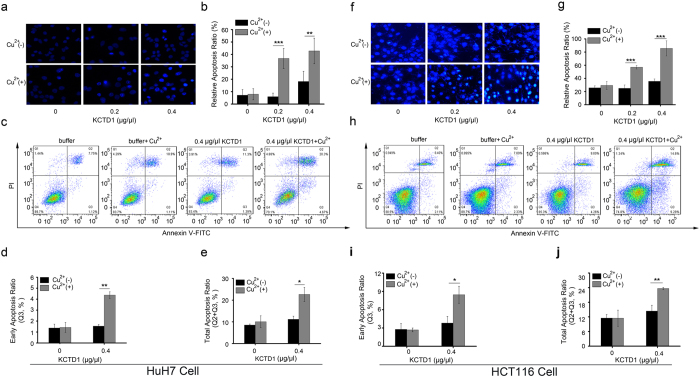
KCTD1 aggregates formed in the presence of Cu^2+^ induces more apoptosis than those formed in the absence of Cu^2+^ in HuH7 and HCT116 cell. (**a**,**f**) Apoptosis was determined by observing chromatin condensation and fragment staining using Hoechst 33258 DNA staining. (**b**,**g**) Apoptotic nuclei were counted as a percentage of total nuclei in Hoechst 33258 DNA staining. (**c**,**h**) Typical images evaluated by flow cytometry. (**d**,**i**) The early apoptosis ratio and (**e**,**j**) total apotosis ratio (early apoptosis and late apoptosis) was quantified by Flow J software. The data shown are the mean ± s.d. of 3 independent experiments. *P < 0.05; **P < 0.01; ***P < 0.001; t-test.

One mechanism for pathogenesis resulting from protein misfolding is due to cytotoxicity. To determine the effect of Cu^2+^-induced KCTD1 misfolding on cell viability, we performed the CCK-8 assay using the human HuH7 cell line. The addition of KCTD1 fibrillar aggregates formed in the presence of 20 μM CuSO_4_ to HuH7 cells caused much more severe cytotoxicity than that caused by KCTD1 aggregates formed without Cu^2+^ (Fig. 7b). To exclude possible Cu^2+^ alone effect on cell, the cytotoxicity of buffer with or without 20 μM CuSO_4_ on HuH7 cells was monitored with the same method, and the result indicated that the cytotoxicity had no relationship with Cu^2+^ (Fig. 7a). The CCK-8 result indicated KCTD1 fibrillar aggregates could decrease cell proliferation strongly, then we considered whether the cell cytotoxic was affected by the cell apoptosis. We performed the Hoechst33258 staining on HuH7 cells and HCT116 which were treated by KCTD1 protein with or without 20 μM CuSO_4_. The result showed that the KCTD1 fibrillar aggregates with 20 μM CuSO_4_ induced more severe apoptosis than that induced by KCTD1 aggregates formed free CuSO_4_, and apoptosis increased in a concentration-dependent manner (Fig. 8a–b,f–g). What’s more, to further obseve the apoptotic cells induced by different treated KCTD1 protein, Annexin-V and PI dual stained HuH7 and HCT116 cells treated with or without 20 μM Cu^2+^ were analyzed by Flow Cytometry, the same results as Hochest33258 have validated that 20 μM Cu^2+^ induced fibrillar aggregates of KCTD1 surely contributed to no matter cell early apoptosis or cell total apoptosis (early apoptosis and late apoptosis) on HuH7 cell lines (Fig. 8c–e)and HCT116 cells (Fig. 8h–j). The cell cytotoxic results suggested that Cu^2+^ affected not only KCTD1 structure, but also its function.

## Discussion

Protein misfolding and aggregation is associated with a wide range of diseases. Several degenerative diseases are associated with prion-like proteins with similar properties as mammalian prion proteins. Although prion diseases are rare, the incidence of prion-like pathologies is high. Metal ions significantly enhance protein misfolding and aggregation, and are directly linked with protein misfolding diseases. Copper ions strongly promote amyloid formation in a variety of proteins. For example, Cu^2+^ binding causes structural changes in prion proteins and has been implicated in prion misfolding and fibril formation^5^,^23^. Proteins self-assemble under increasing Cu^2+^ ion concentrations, resulting in the formation of amyloid fibrils that are cytotoxic^12^.

This study showed that increasing Cu^2+^ concentrations enhanced KCTD1 aggregation. KCTD1 aggregation was first determined by performing turbidity assays. Fluorescence emission assays using ANS and far-UV CD studies detected an increase in β-sheet structure that was dependent on KCTD1 and Cu^2+^ concentration. The KCTD1 structural change indicated that Cu^2+^ enhanced exposure of hydrophobic surface. The ThT spectra suggested that increasing Cu^2+^ concentrations induced formation of fibrillar KCTD1 structures, which was confirmed by transmission electron micrographs.

KCTD1 contains highly conserved BTB domains that mediate protein-protein interactions. KCTD may act as an adaptor for the interactions between the Cul3 ubiquitin ligase, which is involved in protein ubiquitination/degradation, and its substrates. Zhang *et al*. confirmed KCTD1 interacted with beta-catenin and enhanced the ubiquitination/degradation of beta-catenin in a concentration-dependent manner in HeLa cells. And our previous study verified that KCTD1 interacted with PrPC and suggested that KCTD1 could mediate PrP ubiquitination and degradation. Copper accumulates in amyloid plaques in neurological disorders^24^, and KCTD1 is strongly expressed in brain cells^20^. *In vitro* studies, excessive Cu^2+^ would affect KCTD1 structure and lead to KCTD1 aggregation, which might be cause disease. Our study identified a potential mechanism for Cu^2+^-mediated effects on human physiology and pathology through the formation of KCTD1 fibrils.

## Materials and Methods

### Cloning

Construction of the expression vector pET30a-KCTD1 and protein purification were performed as described previously^25^. The eluted protein was desalted with PD-10 desalting columns (GE Healthcare, USA) and stored in 20 μM PBS (pH 7.0) at 4 °C. Protein concentration was measured with a Bio-Rad protein assay kit (Bio-Rad Laboratories, Hercules, CA, USA).

### Secondary structure analysis by far-UV circular dichroism

Far-UV (190–260 nm) circular dichroism (CD) measurements were performed on a chiroptical spectrometer (Jasco, Tokyo, Japan) at 25 °C. The KCTD1 protein sample used for CD measurements had a concentration of 0.4 μg/μl in 20 μM PBS. Measurements were performed with a 1-mm path-length cuvette at 1-nm intervals. Multiple scans were averaged and the background spectra (buffer alone) were subtracted to produce the collated spectrum. The ellipticity of the far-UV CD spectrum was analyzed using DICROPROT software (ICBP, CNRS, Lyon University, Lyon, France).

### Fluorescent dye binding and emission intensity assays

The 5 mM thioflavin T (ThT; Sigma-Aldrich, St. Louis, MO, USA) and 8-anilino-1-naphthalenesulfonic acid (ANS; Sigma-Aldrich) stock solutions were prepared in PBS and filtered with 0.22 μm filters (Merck Millipore, Germany). KCTD1 sample was incubated for 2 h at 37 °C with or without CuSO_4_ in 20 μM PBS. Then, the samples were incubated with either 10 μM ThT or 5 μM ANS in the dark for 10 min at room temperature before measurements respectively. The emission spectra of ThT and ANS were recorded after excitation at 443 nm (excitation and emission slit widths, 5 nm) and 380 nm (excitation and emission slit widths, 10 nm) by Fluorescence spectrophotometer F-4500 (HITACHI, Japan), respectively. Each emission spectrum was the average of three scans. Background fluorescence was subtracted from the results.

### Turbidity measurement

Turbidity was measured to assess protein aggregation. Turbidity measurements were performed at 37 °C in flat-bottomed 96-well plates. The KCTD1 sample in PBS (pH 7.0) was first centrifuged at 12,000 rpm for 5 min at 4 °C, and the supernatant was transferred into a new tube. Then, 200 μl PBS (pH 7.0) containing different KCTD1 concentrations (0.156–5.0 μg/μl) was added to each well in a 96-well plate, and 2 mM CuSO_4_ was added to the sample so the final CuSO_4_ concentration was 20 μM. The samples were mixed immediately in the wells. The sample turbidities were monitored within 5 min by reading the absorbance at 420 nm in a Synergy H1 microplate reader (BioTek, USA) using a kinetic photometric assay protocol (interval time 5 min, 16 cycles with 1 s shaking before every cycle).

### Transmission Electron Microscopy

Electron micrographs were acquired with H-8100 transmission electron microscope (Hitachi, Tokyo, Japan) operated at an acceleration voltage of 100 kV. The aggregation of KCTD1 with or without 20 μM CuSO_4_ was prepared. The protein samples were placed on a carbon-coated copper grid and negatively stained with 2% aqueous phosphotungstic acid for 5 min.

### Proteinase K digestion

Samples were prepared for PK digestion as follows: 0.8 μg/μl KCTD1 with or without 20 μM CuSO_4_ was heated to 37 °C for 2 h in 20 μM PBS (pH 7.0), then the solution was equilibrated at room temperature (25 °C). 0.8 μg/μl BSA samples (as controls) with or without 20 μM CuSO_4_ were dealed with in the same way. The samples were digested with various concentrations of PK (0–20 μg/ml) for 35 min at 37 °C. Digestion was quenched with 2 mM phenylmethanesulfonyl fluoride (PMSF). Protein digestion was evaluated by SDS-PAGE with a 15% polyacrylamide gel in Tris-glycine buffer (pH 8.3) containing 0.1% (w/v) SDS, followed by staining with Coomassie Brilliant Blue.

### Cell cultures

Human Huh7 cells obtained from China Center for Type Culture Collection (CCTCC) were cultured in minimum essential medium (Hyclone, USA) supplemented with 10% fetal bovine serum (Gibco, USA). Cultured cells were grown at 37 °C in a humidified atmosphere containing 5% CO_2_.

### Cell proliferation assay

Cells were plated onto a 96-well plate at a density of 4,000 cells per well in 200 μl of fresh minimum essential medium containing 10% fetal bovine serum and incubated for 24 h at 37 °C in a humidified atmosphere containing 5% CO_2_. 0.8 μg/μl KCTD1 with or without 20 μM CuSO_4_ was heated to 37 °C for 2 h in 20 μM PBS (pH 7.0), Then, 50 μl aliquots of the KCTD1 samples were transferred to wells containing 150 μl fresh Dulbecco’s modified Eagle medium (DMEM) with fetal bovine serum. the control sample were 50 μl PBS (20 μM, pH 7.0). Cell viability of these cultures and controls were evaluated 24 or 48 h later using the cell counting kit-8 (CCK-8) reduction assay. Briefly, 10 μl of CCK-8 (Beyotime Institute of Biotechnology, Haimen, Jiangsu, China) was added to each well and incubated for 1 h at 37 °C. The production of blue formazan crystals was determined by spectrophotometric measurement of absorbance values at 450 nm.

### Cell apoptosis assay

Place a sterile coverslip on the 6 well plate, and seed Huh7 cells or HCT116 on the plate to 80% confluency. Different concentrational KCTD1 (0, 0.8, 1.6 μg/ μl) with or without 20 μM CuSO_4_ was heated to 37 °C for 2 h in 20 μM PBS (pH 7.0), Then, 500 μl aliquots of the KCTD1 samples were transferred to wells containing 1500 μl fresh Dulbecco’s modified Eagle medium (DMEM) with fetal bovine serum. After culturing for 36 hours, the cells were subsequently fixed, washed three times with phosphate buffered saline (PBS), and stained with Hoechst 33258 (Sigma-Aldrich) according to the manufacturer’s protocol. Apoptotic features were assessed by analyzing chromatin condensation and by staining the fragments under an inverted fluorescent microscope (Olympus, Tokyo, Japan).

### Flow Cytometry Assay

Flow cytometry assay was also used to quantify the survival, necrosis and apoptosis of HuH7 and HCT116 cell line. And the cells were treated as previously described as Hochest33258, After culturing for 36 hours, the cells were collected, washed with PBS for three times. And the cells were then filtered with FACS used filter method, then Annexin V-FITC and PI (BD Pharmingin, USA) were used to stain the cells according to manufacture instruction. The cells were then analyzed by FAC-Scan flow cytometry (BD FACSAriaIII, USA), the apoptosis ratio was quantified by Flow J software. The above experiment was repeated three times.

## Additional Information

**How to cite this article**: Liu, Z. *et al.* Bivalent Copper Ions Promote Fibrillar Aggregation of KCTD1 and Induce Cytotoxicity. *Sci. Rep.* **6**, 32658; doi: 10.1038/srep32658 (2016).
